# Preoperative exclusive enteral nutrition ameliorates CRP-to-albumin ratio without affecting body mass index in Crohn’s disease

**DOI:** 10.3389/fnut.2026.1780773

**Published:** 2026-05-29

**Authors:** Jieyi Wang, Yu Shen, Huaying Liu, Zhicheng Wang, Weilin Qi, Wei Liu, Linna Ye, Qian Cao, Xiaolong Ge, Wei Zhou

**Affiliations:** 1 Department of General Surgery, School of Medicine, Sir Run Run Shaw Hospital, Zhejiang University, Hangzhou, China; 2 Inflammatory Bowel Disease Center, School of Medicine, Sir Run Run Shaw Hospital, Zhejiang University, Hangzhou, China; 3 Department of Nutrition and Food Safety, Zhejiang Provincial Center for Disease Control and Prevention, Hangzhou, China; 4 Department of Medicine, Guangxi Health Science College, Nanning, China; 5 Department of Gastroenterology, School of Medicine, Sir Run Run Shaw Hospital, Zhejiang University, Hangzhou, China

**Keywords:** body mass index, C-reactive protein-to-albumin ratio, Crohn’s disease, exclusive enteral nutrition, preoperative optimization

## Abstract

**Background:**

Crohn’s disease (CD) frequently exhibits malnutrition and systemic inflammation, both of which are closely linked to elevated surgical risks and postoperative complications. Exclusive enteral nutrition (EEN) is a standard nutritional therapy for CD, yet whether its core mechanism functions via nutritional support to improve body mass index (BMI) or via nutritional treatment to modulate inflammation, altering the C-reactive protein-to-albumin ratio (CAR) remains inadequately understood. Besides its related alterations in computed tomography enterography (CTE) parameters and laparoscopic surgery outcomes also remain important points.

**Methods:**

A cohort study was conducted at our Inflammatory Bowel Disease Center, enrolling 89 CD patients undergoing bowel resection. Those patients received EEN for 6 weeks preoperatively. All data were collected from a database. Moreover, cross-sectional imaging metrics were assessed by CTE parameters to further demonstrate the impact brought by EEN. Postoperative complications within 30 days were also recorded.

**Results:**

Preoperative EEN treatment significantly increased albumin levels (from 34.4 ± 4.1 g/L at baseline to 38.9 ± 4.5 g/L before surgery, *p* < 0.001), as well as improving inflammatory status via reduced CRP levels [from 23.0 (10.1, 36.5) mg/L at baseline to 2.8 (1.2, 5.0) mg/L before surgery, *p* < 0.001] and consequently decreased CAR (from 0.83 ± 0.79 g/L at baseline to 0.28 ± 0.75 g/L before surgery, *p* < 0.001). Patients receiving EEN also experienced fewer postoperative complications and a shorter postoperative length of stay. However, it was worth noting that BMI remained relative unchanged after EEN therapy (from 19.2 ± 3.4 kg/m^2^ at baseline to 19.3 ± 3.1 kg/m^2^ before surgery, *p* = 0.821).

**Conclusion:**

Preoperative EEN in CD patients effectively ameliorates the CAR without prominently affecting BMI. As a targeted anti-inflammatory strategy in the immediate preoperative period, EEN exerts its benefits primarily through rapid modulation of inflammation rather than weight gain.

## 1 Introduction

Crohn’s disease (CD) is a chronic and relapsing inflammatory disorder of the gastrointestinal tract that can induce transmural inflammation, segmental involvement, and a tendency to develop complications, especially for those genetically susceptible individuals (1). Regarding the rising global incidence of CD, approximately 75% of the patients with CD will inevitably undergo surgery intervention without effective treatment (2). A postoperative surveillance reveals disease activity-driven re-resection rates of 3.6, 10.1, and 14.1% at 1, 5, and 10 years, respectively (3). It indicates that while biologic agents are extensively utilized postoperatively, their therapeutic efficacy remains relative limited showing protective effects only in specific phenotypes (B2/B3) (3), accounting for the merely incremental reduction in overall surgical intervention rates compared to previous studies (4).

Laparoscopic surgery is the preferred first-line approach for CD abdominal surgery, with robust clinical evidence showing superior advantages over open procedures, involving reduced postoperative complications, alleviated pain, faster recovery, and long-term benefits such as decreased adhesion formation and lower incisional hernia rates, as strongly recommended by the European Crohn’s and Colitis Organization (ECCO) 2024 Guidelines (5). It also aligns with enhanced recovery protocols and achieves comparable long-term outcomes while alleviating economic burden compared with biologic agents, making it an alternative option, especially for those ileocolic or limited patients (6).

However, CD patients often face challenges including large inflammatory mass, thickened mesentery, bowel fibrosis, adhesions, pelvic sepsis with fistulizing disease, and malnutrition, which increase surgical difficulty and the risk of conversion, complications, and recurrence (7). The prevalence of malnutrition status is approximately ranging from 12 to 85% in patients with CD (8, 9), as it has become a well-established risk factor for postoperative complications and subsequent body alterations (10). As a result, preoperative optimization to improve nutritional status and reduce inflammation is essential. Exclusive enteral nutrition (EEN) still maintains primary optimization strategy for CD patients requiring surgery. The C-reactive protein-to-albumin ratio (CAR), a novel composite marker derived from C-reactive protein (CRP) and albumin (ALB), has been validated as a prognostic tool in various inflammatory and autoimmune diseases, with higher CAR values correlating with worse outcomes (11, 12). However, the specific effects of preoperative EEN on CAR in CD patients remain underexplored.

In addition to clinical and biochemical markers, cross-sectional imaging, particularly computed tomography enterography (CTE), serves as a valuable non-invasive complement (13). Prior studies have demonstrated that CTE parameters correlate significantly with endoscopic and histological disease activity, and can be used to monitor response to medical therapies (14). It enables visualization of bowel wall thickness, mural healing, stratification, comb sign, and contrast hyper-enhancement, providing objective assessment of intestinal inflammation and structural change in CD (13, 15). Therefore, integrating CTE assessment with biomarkers such as CRP and BMI provides a more comprehensive evaluation of EEN’s preoperative effects, particularly in terms of its potential to improve surgical outcomes through both anti-inflammatory mechanisms and structural healing.

Previous evidence had demonstrated that EEN not only can improves nutritional status but also alleviates intestinal inflammation. Accordingly, we performed a retrospective analysis of CD patients who received preoperative EEN in our Inflammatory Bowel Disease (IBD) center. We focused on changes in CAR, body mass index (BMI), CT-based assessments of intestinal inflammation and structural alterations, and their associations with postoperative outcomes. The primary objective of this study was to investigate whether preoperative EEN influenced BMI and CAR levels.

## 2 Materials and methods

### 2.1 Study design and population

This retrospective study was conducted at our IBD center, involving 89 consecutive CD patients received EEN without other food for 6 weeks preoperatively from January 2015 to December 2020 with a documented plan for future surgery for structuring or penetrating complications of CD. No oral foods or fluids (except for water and weak tea) were allowed. Baseline demographic, clinical, and laboratory parameters were obtained from the institutional database. The CAR, BMI, and inflammatory and nutritional biomarkers were assessed before and after EEN therapy. Postoperative morbidity within the initial 30-day period was meticulously documented.

Inclusion criteria were: (1) diagnosis of CD confirmed by endoscopy, histopathology, and imaging according to the ECCO guidelines; (2) age 18–80 years; (3) preoperative EEN treatment for 6 weeks by exclusive tube feeding, with a daily caloric intake of 30–35 kcal/kg; (4) the score of Nutritional Risk Screening (NRS2002) was more than 2; (5) a confirmed diagnosis of malnutrition according to the European Society for Parenteral and Enteral Nutrition (ESPEN) consensus statement (16); (6) a Crohn’s Disease Activity Index (CDAI) of > 150; and (7) receiving preoperative optimization with EEN which was decided by a multidisciplinary team meeting and also according to the ECCO guidelines. Exclusion criteria were: (1) concurrent malignancy, active infection (excluding CD-related inflammation), or other systemic diseases affecting CRP/ALB levels; (2) preoperative use of parenteral nutrition; (3) incomplete medical records or extensive multi-organ resection; (4) allergic reactions against EN; (5) poor compliance with EEN therapy by tube feeding, like intestinal obstructions or severe shock, or patients with high-output fistulas when receiving EEN (16); and (6) participant in another clinical trial.

### 2.2 Data collection

Baseline demographic data encompassed sex, age, BMI, comorbidities, smoking history, disease duration, CDAI score, preoperative medication history, surgical indications, and Montreal classification, documenting prior to EEN initiation. Laboratory assessments comprised CRP, ALB, and CAR. The CAR was calculated as CRP (mg/L) divided by ALB (g/L), expressed in mg/g. All laboratory tests above, along with critical CTE parameters, were meticulously recorded before and after EEN treatment. Postoperative outcomes in CD patients receiving EEN were also involved.

### 2.3 EEN protocol

All patients received EEN as the sole nutritional source for 6 weeks preoperatively. During this period, no pharmacological treatment was given to these CD patients before surgery for preoperative optimization. The EEN utilized in our current study was Enteral Nutritional Suspension (TPF) (Nutricia Pharmaceutical, Wuxi, China), a polymeric formula with an energy density of 1.5 kcal/mL and an osmolarity of 300 mosm/L. Per 100 mL, it mainly supplied 18.5 g carbohydrates, 6.0 g protein, 5.84 g fat, and 1.5 g dietary fiber. The EEN was administered via tube feeding (like nasogastric tube). The daily caloric intake was individualized to 30–35 kcal/kg actual body weight, with protein accounting for 15–20% of total calories (17).

### 2.4 Surgical procedures and postoperative outcomes

All procedures were performed by experienced surgeons using standard techniques at IBD center. Postoperative complications were monitored for 30 days and classified as mild complications (Clavien-Dindo grade I–II) and major complications (Clavien-Dindo grade III–IV) (18). Postoperative length of stay (LOS) was also recorded (19).

### 2.5 Statistical analysis

Data were analyzed using SPSS 21.0 (IBM Corp, Armonk, NY). Continuous variables are expressed as mean ± SD or median (range) based on distribution, and categorical variables as n (%). Normality was assessed, and group comparisons were made using the *t*-test (normal data) or Mann–Whitney U test (non-normal data) for continuous variables, and the chi-square test or Fisher’s exact test for categorical variables. Univariate analyses compared the CAR, BMI, and other variables between patients before and after preoperative EEN treatment. Spearman’s correlation was used in the analysis. Variables with *p* < 0.05 were entered into multivariate logistic (or linear) regression to identify independent predictors. A *p* < 0.05 was considered statistically significant.

## 3 Results

### 3.1 Baseline characteristics of the study population

A total of 89 CD patients undergoing bowel surgery were included (male: female = 59: 30; mean age 35.6 ± 13.2 years). The mean BMI was 19.2 ± 3.4 kg/m^2^. The mean CDAI score was 249.8 ± 73.6 before EEN. According to the Montreal classification, most patients were diagnosed in adulthood (A2: 60/89, 67.4%; A3: 29/89, 32.6%), with ileal involvement (L1: 68/89, 76.4%) being the most common. Disease behavior was predominantly stricturing (B2: 40/89, 44.9%) or penetrating (B3: 24/89, 27.0%), with 25 patients (28.1%) having combined stricturing and penetrating lesions (B2 + B3). Common surgical indications encompassed ileus (41/89, 46.1%) and fistulas (48/89, 53.9%). Preoperative medications included 5-ASA (28/89, 31.5%), immunomodulators (14/89, 15.7%), biologics (23/89, 25.8%), and corticosteroids (1/89, 1.1%). The clinical characteristic details are demonstrated in Table 1.

**Table 1:** Characteristics of the study population.

Clinical variable	*N* = 89
Male/female	59/30
Age (years)	35.6 ± 13.2
BMI (kg/m^2^)	19.2 ± 3.4
Comorbidities	
Diabetes mellitus	2 (2.2)
Hypertension	1 (1.1)
Smoking	4 (4.5)
Disease duration (months)	24 (5.66)
Preoperative medical therapy	
None	23 (25.8)
5-ASA	28 (31.5)
Systemic steroids	1 (1.1)
Immunomodulators	14 (15.7)
Biologics	23 (25.8)
Surgical indication	
Ileus	41 (46.1)
Fistulas	48 (53.9)
Montreal classification	
Age (years)	
A1 (≤16)	0
A2 (17–40)	60 (67.4)
A3 (>40)	29 (32.6)
Location	
L1	68 (76.4)
L2	0
L3	21 (23.6)
L4	0
Behavior	
B1	0
B2	40 (44.9)
B3	24 (27.0)
B2 and B3	25 (28.1)
Perianal disease	46 (51.7)

BMI, body mass index; ASA, aminosalicylicacid. Data are shown as mean ± SD or median (IQR) for continuous variables, and n (%) for categorical variables.

### 3.2 Changes in biochemical markers and body weight after EEN

As illustrated in Table 2, EEN treatment for 6 weeks contributed to significant enhancements in key inflammatory markers. CRP levels decreased from 23.0 (10.1, 36.5) mg/L (baseline) to 2.8 (1.2, 5.0) mg/L (post-EEN, *p* < 0.001), indicating reduced systemic inflammation. ALB levels rose from 34.4 ± 4.1 g/L to 38.9 ± 4.5 g/L (*p* < 0.001), reflecting improved nutritional status. The CAR levels significantly declined from 0.83 ± 0.79 to 0.28 ± 0.75 (*p* < 0.001). After EEN, the mean CDAI score was decreased significantly (249.8 ± 73.6 vs. 95.2 ± 16.4, *p* < 0.001). However, BMI remained relatively stable (19.2 ± 3.4 kg/m^2^ vs. 19.3 ± 3.1 kg/m^2^, *p* = 0.821), indicating no significant change in overall body weight. Furthermore, Spearman’s correlation analysis showed that CRP and BMI exhibited insignificant correlations (*r* = −0.002, *p* = 0.983).

**Table 2:** Clinical assessment in CD patients with EEN.

Clinical variable	Before EEN (*n* = 89)	After EEN (*n* = 89)	*P*-value
BMI (kg/m^2^)	19.2 ± 3.4	19.3 ± 3.1	0.821
CDAI	249.8 ± 73.6	95.2 ± 16.4	<0.001
CRP (mg/L)	23.0 (10.1, 36.5)	2.8 (1.2, 5.0)	<0.001
ALB (g/L)	34.4 ± 4.1	38.9 ± 4.5	<0.001
CAR	0.83 ± 0.79	0.28 ± 0.75	<0.001

CD, Crohn’s disease; EEN, exclusive enteral nutrition; BMI, body mass index; CRP, C-reactive protein; ALB, albumin; CAR, C-reactive protein to albumin ratio; CDAI, Crohn’s Disease Activity Index. Data are shown as mean ± SD or median (IQR) for continuous variables.

### 3.3 Alterations in CT Enterography parameters

As shown in Table 3, preoperative EEN induced significant radiological improvements in Crohn’s disease (CD) activity, as evidenced by CTE parameters. Specifically, the mean bowel wall thickness (BWT) decreased markedly from 12.2 ± 4.1 mm before EEN to 9.6 ± 3.2 mm after EEN (*p* < 0.001). Meanwhile, mural stratification, defined radiologically as a bilaminar or trilaminar configuration of the small-bowel wall (20), was observed in 79 patients (88.8%) before EEN and decreased to 62 (70.0%) following EEN treatment (*p* = 0.002), suggesting a treatment-related effect. Conversely, the comb sign, characterized by multiple tubular, tortuous opacities aligned like comb teeth on the mesenteric ileum (21), remained highly prevalent, dropping slightly from 86 (96.6%) to 82 (92.1%), *p* = 0.193. Additionally, affected bowel wall attenuation, decreased from 1.8 ± 0.4 HU to 1.6 ± 3.2 HU (*p* = 0.018). Most notably, the CTE decreased significantly from 39.3 ± 9.8 to 31.7 ± 8.7 (*p* < 0.001), further supporting an overall reduction in disease activity as assessed by CTE.

**Table 3:** CT enterography parameters in CD patients receiving EEN before surgery.

Characteristic	Before EEN	After EEN	*P*-value
Bowel wall thickness (mm)	12.2 ± 4.1	9.6 ± 3.2	<0.001
Mural stratification	79 (88.8)	62 (70.0)	0.002
Comb sign	86 (96.6)	82 (92.1)	0.193
Affected bowel wall attenuation (HU)	1.8 ± 0.4	1.6 ± 3.2	0.018
CTEIA	39.3 ± 9.8	31.7 ± 8.7	<0.001

CT, computed tomography; CD, Crohn’s disease; EEN, exclusive enteral nutrition; CTEIA, CT enterography index of activity. Data are shown as mean ± SD for continuous variables, and n (%) for categorical variables.

### 3.4 Postoperative outcomes

Among the 89 patients, 75 (84.3%) explored an uneventful recovery, while 14 (15.7%) encountered postoperative complications. As demonstrated in Table 4, mild complications (Clavien-Dindo I–II) occurred in 11 patients (12.4%), including bowel obstruction (7/89, 7.9%), wound infection (3/89, 3.4%), and low-grade fever (1/89, 1.1%). Simultaneously, major complications (Clavien-Dindo III–IV) were observed in 9 patients (10.1%), comprising gastrointestinal bleeding, anastomotic leakage, intra-abdominal abscess, and intra-abdominal bleeding, which accounted for 3.4, 3.4, 2.2, and 1.1%, respectively. Furthermore, compared with the previous study conducted by our hospital’s IBD center, the rate of postoperative complications in the current EEN group was 22.5%, which was significantly lower than that in the non-EEN group (36.0%) (22). The median postoperative LOS was 7 days (IQR: 7–8 days), demonstrating a prominent decrease than the non-EEN group (10.3 ± 0.7 days) (22). No EEN-related adverse events, such as tube dislodgement and severe diarrhea, were reported.

**Table 4:** Postoperative outcomes analysis after surgery in CD patients receiving EEN.

Postoperative complications	All (*n* = 89)	Postoperative complications	All (*n* = 89)
Postoperative length of stay	7 (7,8)	Major complications	9 (10.1)
Mild complications	11 (12.4)	Intra-abdominal bleeding	1 (1.1)
Bowel obstruction	7 (7.9)	Gastrointestinal bleeding	3 (3.4)
Wound infection	3 (3.4)	Intra-abdominal abscess	2 (2.2)
Fever >38.5 °C after surgery	1 (1.1)	Anastomotic leakage	3 (3.4)

CD, Crohn’s disease; EEN, exclusive enteral nutrition. Data are shown as median (IQR) for continuous variables, and n (%) for categorical variables.

## 4 Discussion

In this retrospective study, we evaluated whether preoperative EEN could improve inflammatory and biochemical improvement status and structural status through CT scan, thereby optimizing surgical outcomes in patients with CD undergoing bowel resection. The study demonstrated that patients who received 6 weeks of preoperative EEN exhibited significant reductions in inflammatory status and improved structural status according to CTE parameters, with a significant decrease in the CAR. These findings align with those of Ge et al., who reported that EEN significantly improved biochemical improvement status and reduced inflammatory markers, without weight gain, subsequently enhancing patients’ physiological status for surgical intervention and recovery (22).

Although both CRP and ALB are predominantly synthesized in the liver (23), CRP functions as an acute-phase reactant that promptly responds to inflammation triggered by tissue trauma or infectious processes (23, 24), and serves as a key biomarker for assessing systemic inflammatory processes with broad clinical utility (11), whereas ALB is essential for sustaining metabolic stability and nutritional balance (25). Given that each biomarker exhibits unique performance attributes and inherent limitations, their combined application can compensate for individual deficiencies and synergistically improve diagnostic precision (26). Accordingly, in the present study, we adopted the composite CAR to systematically investigate the mechanistic role of EEN.

Furthermore, elevated CAR, typically indicative of high systemic inflammation coupled with poor nutritional status, has been validated as a prognostic indicator in inflammatory status (11, 12), as well as an independent risk factor for the activity and severity of CD patients (27). Extending its clinical relevance, Zhou et al. documented the unique role of CAR in predicting mucosal healing in Crohn’s disease (CD), demonstrating an excellent discriminatory power in inflammatory diseases (28). In our present study, integrated with prior evidence above, this EEN-induced CAR reduction not only objectively validates the therapy’s capacity to concurrently suppress systemic inflammation and correct malnutrition but also serves as a prognostic biomarker, wherein lower post-treatment CAR values are associated with sustained clinical remission and a reduced risk of long-term complications.

Although EEN is an established and effective therapy, its therapeutic response is typically monitored using clinical, biochemical, and endoscopic assessments (29). While these multidimensional assessments provide comprehensive insights into EEN response, CTE is rarely utilized in the evaluation of EEN efficacy. Our study addresses this gap by employing CTE to evaluate radiological markers of intestinal inflammation and treatment response, thereby providing novel insights into the effects of EEN. As previously reported, bowel wall thickness, mural stratification, engorged vasa recta (comb sign), and increased bowel wall attenuation are associated with active inflammation (30). The results showed that EEN was associated with significant improvements in key radiological inflammatory markers, particularly bowel wall thickness (BWT) and the computed tomography enterography activity index (CTEIA) (*p* < 0.001). Given that BWT is the most commonly reported and clinically relevant CT feature in patients with CD (31), demonstrating the best diagnostic performance for assessing activity, and that CTEIA functions as a novel qualitative tool designed for evaluating ileocolonic CD activity (32), these metrics provide critical objective evidence together. The imaging-based improvements directly reflect EEN’s efficacy in reducing intestinal inflammation, alleviating bowel wall fibrosis and edema, thereby improving the surgical field and lowering the risks of intraoperative injury and postoperative complications. Furthermore, radiological markers of bowel enhancement on CTE have been shown to be positively correlated with endoscopic and histological severity, as well as serum CRP levels in CD patients, aligning with our current clinical trends and previous results from our center (22).

Epidemiological data from hospitalized CD cohorts reveal a high prevalence of nutritional impairment, with 75% classified as malnourished and approximately one-third presenting with a low BMI, underscoring the critical need for targeted nutritional interventions in this population (33). Notably, despite EEN significantly improving inflammatory and nutritional markers, the patients’ BMI remained within the normal range for BMI throughout the treatment period. BMI is an anthropometric parameter, and no significant change was found after EEN therapy for even 6 weeks. While some prior studies reported EEN-associated increases in body weight or BMI, this discrepancy may stem from differences in study populations, intervention durations, or primary endpoints (34, 35). Our findings align more closely with Ge et al., where BMI was not a predefined focus (22). As an indispensable indicator of overall adiposity and nutritional status (25, 36), BMI may be less responsive to short-term EEN interventions due to limited time for fat or muscle accumulation, particularly in the preoperative phase (37). In addition, this insensitivity is further compounded in active-phase CD patients, whose sustained disease activity disrupts nutrient absorption and accelerates catabolic metabolism, thereby limiting substrate availability for fat or muscle deposition (38). Collectively, the stability of BMI under these conditions does not reflect EEN inefficacy but rather underscores the prioritization of targeted therapeutic goals over anthropometric metrics in short-term nutritional strategies.

Beyond its direct benefits, EEN may attenuate inappropriate reliance on conventional pharmacological therapies in CD, thereby alleviating the burden of treatment-associated adverse effects. In this study, 31.5% of patients received 5-aminosalicylic acid (5-ASA), 25.8% were treated with biologic agents, and 15.7% with immunomodulators. According to the ECCO guidelines, although medical treatments are effective for managing disease activity, they are associated with potential side effects, including infections, organ toxicity, as well as metabolic complications occasionally observed in corticosteroid users (39). EEN may decrease the need for such therapies as a safe and non-drug intervention by promoting mucosal healing, inducing remission, and improving nutritional status, particularly in the preoperative setting, eventually offering a valuable adjunct in the holistic management of CD (39).

Among various surgery options, laparoscopic surgery is the first-line approach for abdominal surgery in CD due to its minimally invasive nature, accelerated recovery, and long-term benefits in decreasing adhesion formation (5). Some studies were reported that the complication rate of laparoscopy group still arrived 43.1% (40), underscoring the need for adjunctive strategies. To further alleviate postoperative complications, our current study showed that patients receiving EEN had a significantly lower overall postoperative complication rate, with a notable reduction in major complications. The median postoperative hospital stay in the EEN group was 7 days, significantly shorter than the 10.3 ± 0.7 days in the historical non-EEN group (22). These findings are highly consistent with Ge et al. (22). Collectively, the evidence, along with our study, positions EEN as a clinically valuable intervention to optimize surgical outcomes in CD.

There were several limitations of our study that must be considered. First and foremost, as a single-center retrospective cohort study, inherent selection bias could not be entirely excluded, concerning that patients with EEN may have had higher baseline compliance or milder disease compared to non-EEN counterparts. Meanwhile, the relatively small sample size may limit statistical power and the reliability of subgroup analyses, like subgroups according to pharmacological treatment. Additionally, the absence of direct EEN vs. non-EEN group comparisons meant no concurrent controls were available; instead, historical controls and multivariate analyses were used to indirectly assess EEN’s effects. Furthermore, all participants received a standardized 6-week EEN intervention, precluding evaluation of alternative duration or personalized regimens, thus constraining the BMI-related finding. Last but not least, the nutritional status was only based on biochemical parameters, without anthropometry or body composition.

## 5 Conclusion

Our study demonstrated that preoperative EEN therapy in CD patients significantly reduced the CAR and concurrently improved CTE parameters, with no significant change in BMI, indicating its primary effect was anti-inflammatory rather than body weight. These anti-inflammatory benefits may further reduce the risk of postoperative complications, supporting the use of EEN as a valuable preoperative strategy in patients undergoing laparoscopic surgery for CD. However, future study may not only include concurrent controls and more information about nutritional status in order to emphasize EEN has greater effects on inflammatory and structural status than on nutritional status, but also enroll more participants for subgroup analysis.

## Data Availability

The raw data supporting the conclusions of this article will be made available by the authors, without undue reservation.
